# Validation of a Portable Device for Mapping Motor and Gait Disturbances in Parkinson’s Disease

**DOI:** 10.2196/mhealth.3321

**Published:** 2015-02-02

**Authors:** Alejandro Rodríguez-Molinero, Albert Samà, David A Pérez-Martínez, Carlos Pérez López, Jaume Romagosa, Àngels Bayés, Pilar Sanz, Matilde Calopa, César Gálvez-Barrón, Eva de Mingo, Daniel Rodríguez Martín, Natalia Gonzalo, Francesc Formiga, Joan Cabestany, Andreu Catalá

**Affiliations:** ^1^Fundación Sant Antoni Abat (Consorci Sanitiari del Garraf)Vilanova i la GeltruSpain; ^2^School of Engineering and Informatics, National University of Ireland at GalwayGalwayIreland; ^3^Technical Research Centre for Dependency Care and Autonomous Living, Universitat Politècnica de CatalunyaVilanova i la GeltrúSpain; ^4^Department of Neurology, Hospital Universitario Infanta CristinaParla, MadridSpain; ^5^Parkinson’s & Movement Disorders Unit, Centro Médico TeknonBarcelonaSpain; ^6^Department of Neurology, Consorci Sanitari del MaresmeMataró, BarcelonaSpain; ^7^Department of Neurology, Hospital Universitari de BellvitgeBarcelonaSpain; ^8^Fundación Sant Antoni Abat (Consorci Sanitiari del Garraf)Vilanova i la GeltrúSpain; ^9^Hospital Universitari de BellvitgeBarcelonaSpain

**Keywords:** accelerometer, kinematic sensor, motor fluctuations

## Abstract

**Background:**

Patients with severe idiopathic Parkinson’s disease experience motor fluctuations, which are often difficult to control. Accurate mapping of such motor fluctuations could help improve patients’ treatment.

**Objective:**

The objective of the study was to focus on developing and validating an automatic detector of motor fluctuations. The device is small, wearable, and detects the motor phase while the patients walk in their daily activities.

**Methods:**

Algorithms for detection of motor fluctuations were developed on the basis of experimental data from 20 patients who were asked to wear the detector while performing different daily life activities, both in controlled (laboratory) and noncontrolled environments. Patients with motor fluctuations completed the experimental protocol twice: (1) once in the ON, and (2) once in the OFF phase. The validity of the algorithms was tested on 15 different patients who were asked to wear the detector for several hours while performing daily activities in their habitual environments. In order to assess the validity of detector measurements, the results of the algorithms were compared with data collected by trained observers who were accompanying the patients all the time.

**Results:**

The motor fluctuation detector showed a mean sensitivity of 0.96 (median 1; interquartile range, IQR, 0.93-1) and specificity of 0.94 (median 0.96; IQR, 0.90-1).

**Conclusions:**

ON/OFF motor fluctuations in Parkinson's patients can be detected with a single sensor, which can be worn in everyday life.

## Introduction

### The ON/OFF Phase Detection

Patients with idiopathic Parkinson’s disease (PD) report fluctuations between an ON-phase—where symptoms are under control—and an often suddenly starting OFF-phase, where many symptoms reappear and their gait turns abnormally slow.

Collecting precise information on the temporal course of OFF episodes (onset and duration) is essential for adjusting a therapy schedule aimed at preventing motor fluctuations in PD patients (ON/OFF changes). The time-in-OFF is the main parameter used to evaluate the effectiveness of a pharmacological intervention and compare the action of different active principles in clinical trials [1].

Currently, the most common method of collecting such information consists in asking the patients to keep an ON/OFF diary. However, this method is rather time consuming for the patient and has a number of limitations, including recall bias and reduced compliance, which make it unsuitable for medium and long term monitoring in clinical practice [2].

Therefore, portable electronic detectors, which could reliably and automatically record patient’s motor fluctuations would be welcome. Patients could wear such devices in their daily life activities, provided that devices were small and autonomous enough. Furthermore, the use of such devices would open the possibility of automatic or semiautomatic real-time control of drug infusion rates in currently available drug-pumps (apomorphine or duodopa pumps). Thus, it would be possible to increase the dose when the beginning of an OFF-phase is detected and to reduce it again by the beginning of a new ON-phase.

Up to the moment, experiments with inertial sensors (mainly accelerometers and gyroscopes) have been conducted with the aim of producing a detector capable of determining whether a patient is in ON or OFF [3-6]. Such experiments, however, were carried out in controlled settings (laboratory), where the patients were asked to perform specific maneuvers (eg, sections of the Unified Parkinson's Disease Rating Scale) while wearing the sensors (generally, several sensors located on different parts of the body). In most of these experiments, OFF periods were artificially induced by prolonged withdrawal of the patients’ habitual medication. The so induced OFF periods are usually deeper and more clearly defined than OFF periods naturally occurring to patients on their habitual medication. Therefore, the suitability of algorithms validated under controlled conditions to detect natural spontaneous OFF-periods in real life situations (medicated patients in their natural environment) cannot be assured.

### The Present Study

The present study was designed as proof of concept for evaluating the feasibility of reliably detecting motor fluctuations in patients performing daily life activities in their habitual environment. The tested device consisted of a unique component attached to the patient’s waist, which was easily portable in real-life conditions. In this article, we report the validity of data corresponding to measurements taken by the tested device.

## Methods

### Design

**Figure 1:**
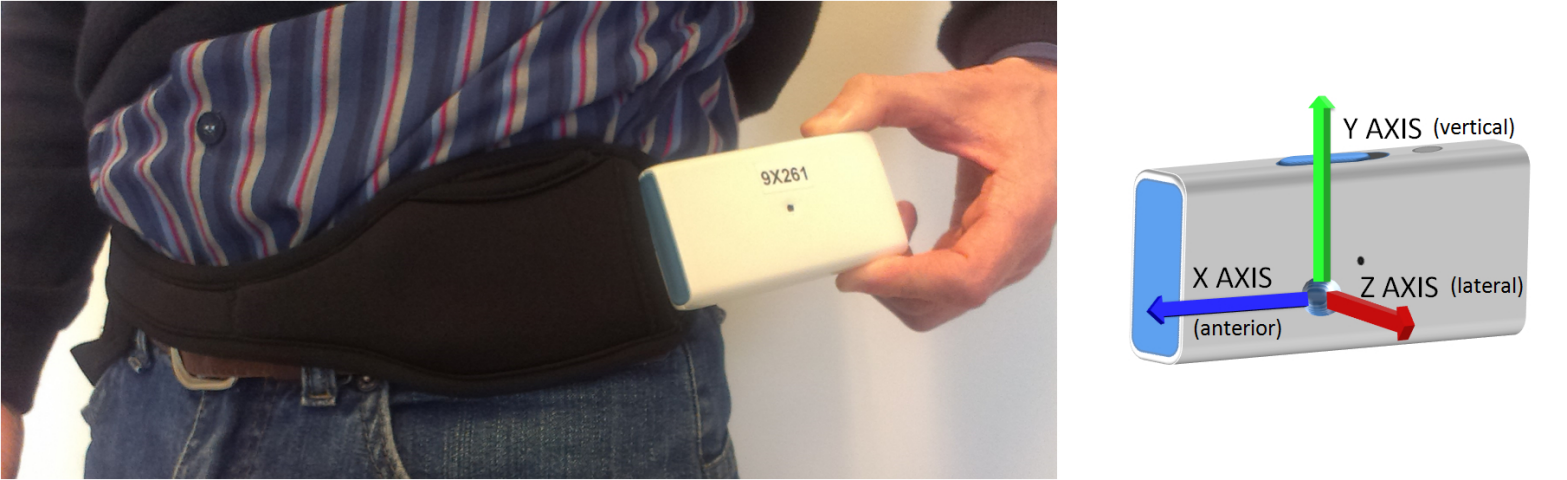
Portable inertial sensor and the neoprene belt where the sensor was inserted.

Algorithms for processing the signals produced by a portable inertial sensor (triaxial accelerometer) designed to detect motor fluctuations (ON/OFF) in Parkinson patients were developed and validated. The study was conducted in two parts. In the first part, an inertial signal database was created with data recorded from 20 patients who were asked to wear the sensor device attached to their waist (Figure 1 shows this device). The algorithms to detect different motor phases were developed using this database. In the second part, the algorithms were validated with data from a new sample of 15 patients (who did not take part in the first part) who were asked to wear the same device.

### Participants and Settings

This study was carried out during 2008 and 2009, with patients between 55 and 75 years of age, living in the Barcelona area, who had been diagnosed to have idiopathic PD according to the criteria of the Brain Bank, London [7]. Only patients with moderate-severe PD and motor fluctuations were included in the second part of the study (while patients with milder forms of the disease, who did not have motor fluctuations, had been allowed to participate in the first part).

Volunteer patients were recruited by convenience sampling among members of local Parkinson patients associations (Associació Catalana per al Parkinson) and patients visiting the Neurology Department of the Consorci Sanitari del Maresme (Barcelona), the Teknon Medical Center (Barcelona), and the University Hospital of Bellvitge (Barcelona).

Participants finally included in the study were 64 years old on average; 27 (77%, 27/35) of them were men and 8 (23%, 8/35) were women. The median number of years of progression of the disease was 9.5 (interquartile range, IQR, 2-18) and the median Hoehn and Yahr score was 3 (IQR 1-4).

The first part of the study was conducted in the facilities of the Universitat Politècnica de Catalunya and two regional hospitals: (1) Consorci Sanitari del Garraf, and (2) Consorci Sanitari del Maresme, all of them located in Barcelona (Spain). The second part was conducted at the patients’ homes.

The Ethical Committee of the Consorci Sanitari del Maresme approved the protocol of the study. All participants signed an informed consent form before their inclusion in the study.

### Procedure for Data Collection

A unique research team (a physician, two nursing assistants, and three engineers) specifically trained in the procedures of the study and the administration of the involved questionnaires collected the data.

In the first part of the study (20 patients), data were collected and used to create processing algorithms for detection of ON/OFF motor fluctuations. A first group of 10 patients participated in an experiment under controlled conditions; they were asked to walk 5 meters straight (3 times) while wearing the inertial sensor device attached to the waist (left side). Patients who presented motor fluctuations repeated this experiment both in ON and OFF. The remaining 10 patients participated in another experiment under controlled and uncontrolled conditions. They were asked to complete a movement circuit in the laboratory (controlled conditions), which included walking straight, walking up and down stairs, walking up and down inclined planes, making turns, taking different positions such as sitting, standing up or lying down, walking while carrying a glass of water, walking while carrying a heavy object, and other more complex activities, such as setting the table for a meal. After the circuit, they were asked to take a 15-minute walk outdoors (uncontrolled conditions). Patients who presented motor fluctuations went through this protocol both in ON and OFF. In this first part of the study, reducing patients’ dopaminergic medication, when necessary, could induce the OFF status.

In the second part of the study, the processing algorithms developed with data from the first part were validated on 15 new patients. These patients were asked to wear the sensor device during 3 to 5 hours while performing their habitual activities in an environment that was familiar to them (home, neighborhood, or habitually visited places). During this validation phase, the OFF status was not induced by modifying patients’ medication schedules; OFF data were collected during naturally occurring OFF episodes. With the aim of increasing the probability of recording at least one naturally occurring OFF episode during the monitoring hours, on the day of the experiment, patients were appointed at the time they typically experienced OFF status.

### Measurement Instruments and Control Variables

The sensor used to record inertial signals is a prototype of an inertial measurement unit developed at the Universitat Politècnica de Catalunya, which includes triaxial sensors to record acceleration in their space frame [8]. The triaxial sensor of lineal acceleration (LIS3LV02DQ, STMicroelectronics) can be used to measure acceleration magnitudes up to ±6 G (1 G=9.81 m/s^2^), with 2.404 mg sensitivity and 2% maximum nonlinearity on the full scale range. The inertial device is “wearable” and equipped with data processing and wireless information transfer capacities and rechargeable batteries. The sensor sampling frequency was 200 Hz. Signals obtained were downsampled to 40 Hz, since this last frequency is enough to obtain 99% of the acceleration power during walking [9].

Video recording was the gold standard used to identify patients’ movements, positions, and ON-/OFF-episodes in experiments under controlled conditions (laboratory) in the first part of the study. Physicians experienced in movement disorders reviewed videos. Additionally, patients were asked to confirm the occurrence of ON-/OFF-episodes. No discrepancies were found between patients’ self-reported motor status and expert diagnosis based on video review.

The gold standard used in experimental procedures conducted under uncontrolled conditions (on the street, at home, etc), both in the first and the second parts of the study, was an observer trained in motor symptoms recognition, who accompanied the patient all the time and recorded the occurrence of ON-/OFF-episodes. Patients were asked to confirm the occurrence of every observed ON-/OFF-episode before recording it. For intermediate status between OFF and ON reported by the patients, the term “intermediate” was used. In case patient and observer disagreed about the motor status, the period was labeled “undefined”. Data were recorded in real-time using a personal digital assistant (ultra mobile personal computer) with software developed by researchers from the Universitat Politècnica de Catalunya. The software required the researcher to update the patient’s motor status every 30 minutes. The researchers in charge of gold standard reading were blind to the results of the algorithms.

After the experimental sessions, patients were administered a specific questionnaire of usability; they were asked about the comfort of wearing the sensor (Likert scale, 1 to 5), possible movement hindrance (yes, no, or slight), preferred location of sensor (waist, legs, or feet), and willingness to wear it daily (open answer). Additionally, by using structured questionnaires, control variables and descriptive data were collected. Such additional data were: (1) sex, (2) age, (3) Hoehn and Yahr stage, (4) year of diagnosis, (5) time of evolution of the disease (in years), and (6) complete list of medicines.

### Signal Processing and Analysis Methods

#### Overview

Inertial data recorded by accelerometers during the first part of the study were used to develop soft-computing techniques aimed at identifying the ON/OFF status. The developed algorithms are described in this section.

The method used to characterize the ON/OFF status has been published elsewhere [10]. The method is based on analyzing patients’ motion fluency while walking and consists of four phases.

#### Phase 1

The first phase is focused on detecting walking periods. The accelerometer signal was represented as the summation of the three axis of spectral power in the [0.1, 3] Hz and [0.1, 10] Hz ranges, contained in 3.2 second windows (128 samples at 40 Hz). These two features were selected among frequency ranges *[b*_*1*_ *, b*_*2*_ *]* that satisfied *b*_*1*_ *,b*_*2*_ε {0.1, 0.2,…, 19.8, 19.9, 20}, *b*_*2*_> *b*_*1*_by means of a ReliefF feature selection algorithm [11]. Both features were used as input for a support vector machine (SVM) [12] whose output was used to classify every window as “walking” or “not walking”. The SVM used a radial basis function kernel, and its parameters C and γ were set through a stratified 10-fold cross-validation process applied to data from first part of the study.

#### Phase 2

The second characterization phase was focused on detecting patients’ strides. The principles described by Zijlstra and Hof [13]—based on the behavior of inertial measurements recorded from the L3 vertebra during gait—were adapted to the location of the sensor in this study. These principles are focused on detection of relative extrema in the forward acceleration signal that are known to correspond to the initial contact event of the gait cycle. Since fragments corresponding to gait initiation and termination were considered irrelevant to detection of “walking” or “not walking”, the first and last window of each detected period were left out of the stride detection analysis.

#### Phase 3

In the third ON/OFF characterization phase, the strides detected during the second phase were evaluated through the spectral power in the [0.1, 10] Hz range. This frequency range was found to maximize ON/OFF discrimination as measured through the area under the receiver operating characteristic curve, in the above-mentioned frequency ranges [*b*_1_, *b*_2_]. The resulting measurement was a representation of a stride, which was proportional to the patient’s motion fluency, the higher the value, the deeper the ON status [14].

#### Phase 4

In the last phase, all fluency values that represented the strides of a same gait episode were averaged (disregarding the two initial and two final strides). The averaged value was compared with a threshold (unique to every patient) to determine the patient’s motor status at that moment. If the averaged measurement was higher than the threshold, the motor status was considered to be ON. Conversely, if the averaged measurement was equal or lower than the threshold, the motor status was considered to be OFF. The threshold was adapted to every individual patient by using 20% of the data available from the patient.

### Statistical Analysis

To assess the validity of the algorithms developed in the first part of the study, they were applied to the inertial signals recorded during the second part. The outcomes of the algorithms were compared with reference standards. Thus, data from participants in the first part—used to create the algorithms—were not used in the validation process.

To assess the validity of the ON/OFF detection algorithm, its results (continuous numerical variable) had to be classified into “ON” or “OFF” categories after establishing a splitting ON/OFF threshold. As described in the above section Signal Processing and Analysis Methods, the value of such threshold is specific for every Parkinson patient and has to be individually established. Thus, although the algorithm was developed in the first part of the study, individual thresholds were established for patients who participated in the validation phase (second part).

To establish the ON/OFF splitting threshold for patients in the second part of the study, measurements describing a patient’s strides were split into two datasets, one of these datasets was used to fix the threshold, while the other dataset was used to assess the validity of the algorithm against that threshold. The first dataset was a randomly selected 20% of measurements consecutively recorded both in ON and OFF. The remaining data were included into the second dataset. All the validity values reported in this article correspond to the analysis of the second dataset. To minimize the effects of arbitrary selection of the data used to establish individual thresholds, the random splitting process was repeated 30 times.

ON/OFF splitting was established through a SVM with a linear kernel. Since the data to be classified were scalar, the splitting hyperplane found during the training process with the first dataset was a scalar value from which the threshold took its value. Therefore, when enough data were available (at least 10 patterns for every class), 10-fold cross validation (CV) was used; otherwise, 2-fold (minimum) CV was used. SVM with linear kernel was used to follow the maximum margin principle that ensures a good generalization since the Vapnik-Chervonenkis dimension was maximized [12].

The sensitivity, specificity, and positive and negative predictive values corresponding to the ON/OFF detection algorithms were calculated. The ON/OFF algorithm was studied by applying it, on the one hand, to all the detected walking segments and, on the other hand, to segments containing 10 or more strides.

There were five minutes of signal, before the start and after the termination of a motor phase, that were excluded from the analysis as they were considered to be in the margin of synchronization error with the gold standard (a time margin was allowed for the patient and the observer to notice and report a change in motor status). Signal segments corresponding to undefined or signal segments lacking comparison standard because of technical errors or artifacts were disregarded. Motor phases defined as intermediate between ON and OFF were also disregarded, since a gold standard for such phenomena is not available (in current clinical practice, ON/OFF is dichotomous concept).

The validity of the algorithms was studied for individual patients and the results were averaged.

## Results

A total of 46.9 hours of inertial sensor signals were recorded, which corresponded to the motion records of the 15 subjects who participated in the validation phase (a mean of 3.1 hours per patient, range 1.4-5.5 hours). The ON/OFF detection algorithm applied to these data yielded valid 1562 results, 1196 of them corresponded to ON (863 of them derived from walking segments of 10 or more strides), 366 corresponded to OFF (267 derived from walking segments of 10 or more strides), and 276 corresponded to intermediate between ON and OFF. The detection algorithm produced an output every 1.09 minutes on average (SD 4.25 minutes; maximum time without producing an output, 70 minutes) for a walking segment. However, when only segments with 10 or more strides were considered, the detection algorithm made a decision every 3.9 minutes on average (SD 10 minutes; maximum time without producing an output, 136 minutes).

For four patients participating in the second part of the study, the recorded motor data (ON/OFF) were not enough to apply the validation method. For the remaining 11 patients, the mean validity values for the ON/OFF detector were, sensitivity 0.91 (median 1; IQR 0.85-1), specificity 0.90 (median 0.92; IQR 0.81-0.92), positive predictive value 0.80 (median 0.80; IQR 0.70-0.95), and negative predictive value 0.94 (median 1; IQR 0.89-1). When only walking segments of 10 or more strides were considered (there were 10 patients with a complete dataset), the mean validity values were, sensitivity 0.96 (median 1; IQR, 0.93-1), specificity 0.94 (median 0.96; IQR 0.90-1), positive predictive value 0.90 (median 0.92; IQR 0.80-1), and negative predictive value 0.98 (median 1; IQR 0.97-1).

Table 1 shows the sensitivity, specificity, and positive and negative predictive values for the ON/OFF detection algorithm. The time the participants spend in the different motor phases (ON/OFF) and the number of walking bouts analyzed is also shown in Tables 1 and 2.

**Figure 2:**
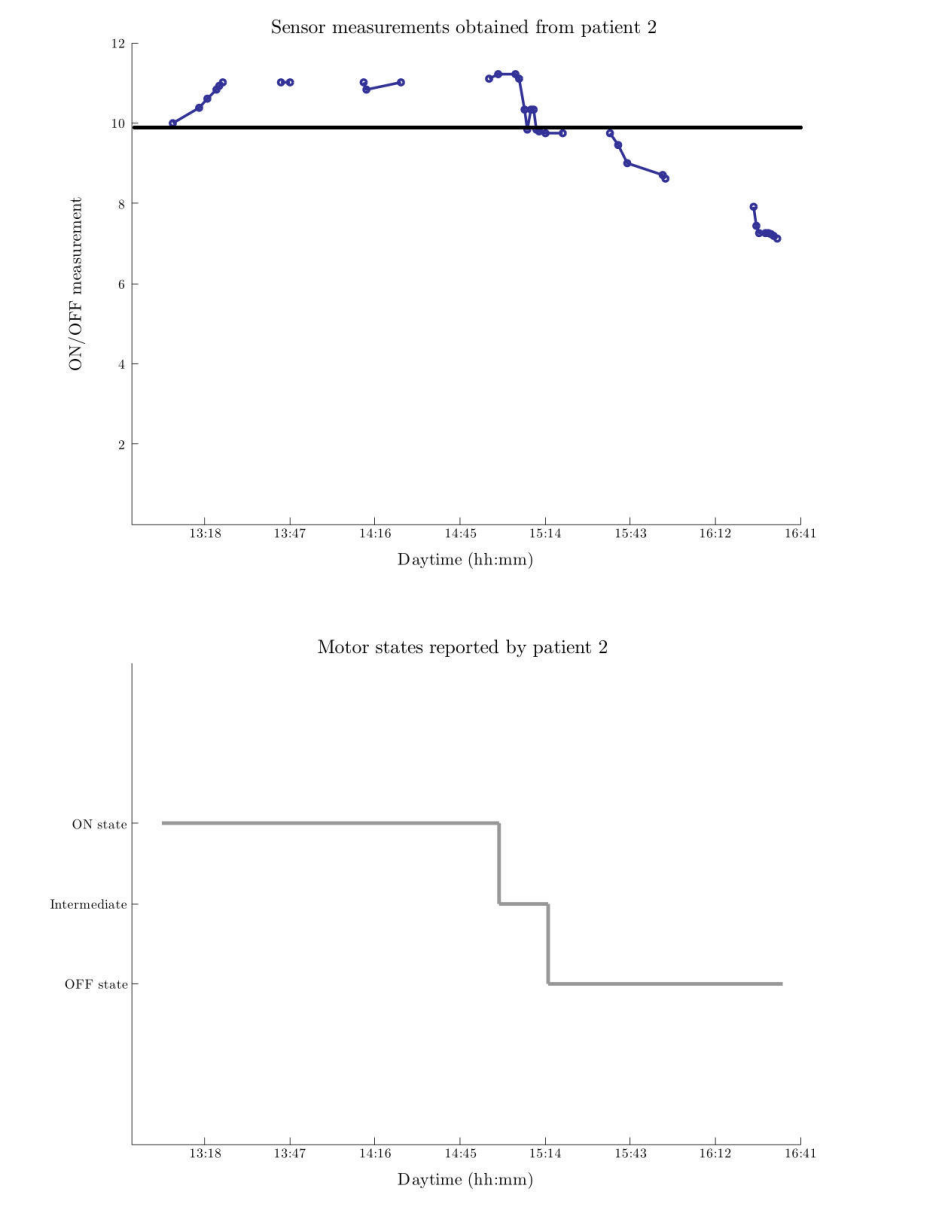
Upper figure shows sensor’s measurements along time for patient 2. Threshold found to separate motor states is also depicted. Lower figure presents the corresponding gold standard, which is the reported motor status according to patient-2 diary. hh:mm = hour and minutes.

Figure 2 shows as an example the results produced by the ON detection algorithms, together with the corresponding motor state (ON/OFF) gold standard for patient number 3. This figure shows the intermittent detection of the ON-OFF phase provided by the sensor (the outcome of the algorithm is a continuous numerical variable), the motor state reported by the participant and verified by an observer (ON/OFF or an intermediate phase), and the threshold found that allows distinguishing both motor states.

All of the participants rated the comfort of wearing the sensor 4 (good) or 5 (very good). None of them reported the sensor to hinder or restrict their activity, and only one patient would refuse wearing it daily. Only one participant preferred the sensor to be located on the leg.

**Table 1:** Algorithm applied to walking segments of any length.

Patient	H&Y^a^	# Segments OFF	# Segments ON	Minutes OFF	Minutes ON	Specificity % (SD)	Sensitivity % (SD)	PPV %^b^(SD)	NPV %^c^(SD)
1	3	86	15	22	122	97 (4)	100 (0)	99 (1)	100 (0)
2	4	23	118	107	161	92 (12)	100 (0)	81 (27)	100 (0)
3	3	5	25	7	213	-	-	-	-
4	3	21	37	26	125	83 (13)	89 (23)	80 (15)	95 (9)
5	2.5	18	97	18	276	99 (1)	100 (0)	95 (3)	100 (0)
6	3	14	120	7	159	94 (4)	100 (0)	70 (2)	100 (0)
7	2.5	25	42	5	159	96 (3)	100 (0)	94 (5)	100 (0)
8	2	15	121	3	138	90 (4)	100 (0)	59 (17)	100 (0)
9	2	5	205	3	327	-	-	-	-
10	4	38	44	11	13	80 (27)	56 (23)	76 (31)	70 (14)
11	1.5	0	50	0	191	-	-	-	-
12	2.5	17	162	22	260	81 (16)	85 (20)	48 (30)	99 (2)
13	3	40	13	41	179	100 (0)	90 (5)	100 (0)	77 (11)
14	2.5	0	60	2	73	-	-	-	-
15	3	59	87	37	107	77 (11)	80 (32)	71 (10)	89 (15)

^a^H&Y=Hoehn and Yahr scale

^b^PPV = positive predictive value

^c^NPV = negative predictive value

**Table 2:** Algorithm applied to walking segments of 10 or more strides.

Patient	H&Y^a^	# Segments OFF	# Segments ON	Minutes OFF	Minutes ON	Specificity % (SD)	Sensitivity % (SD)	PPV %^b^(SD)	NPV %^c^(SD)
1	3	74	14	22	122	95 (5)	100 (0)	99 (1)	100 (0)
2	4	10	84	107	161	94 (3)	100 (0)	68 (15)	100 (0)
3	3	5	5	7	213	-	-	-	-
4	3	8	21	26	125	88 (9)	96 (7)	78 (15)	99 (3)
5	2.5	9	91	18	276	100 (0)	100 (0)	100 (0)	100 (0)
6	3	9	88	7	159	100 (0)	100 (0)	100 (0)	100 (0)
7	2.5	25	41	5	159	96 (0)	100 (0)	94 (4)	100 (0)
8	2	12	114	3	138	97 (4)	100 (0)	81 (21)	100 (0)
9	2	0	160	3	327	-	-	-	-
10	4	28	30	11	13	83 (26)	82 (11)	87 (17)	86 (8)
11	1.5	0	5	0	191	-	-	-	-
12	2.5	5	85	22	260	-	-	-	-
13	3	29	10	41	179	100 (0)	100 (0)	100 (0)	100 (0)
14	2.5	0	45	2	73	-	-	-	-
15	3	53	70	37	107	90 (8)	83 (24)	89 (7)	91 (13)

^a^H&Y=Hoehn and Yahr scale

^b^PPV = positive predictive value

^c^NPV = negative predictive value

## Discussion

### Principal Results

In the present study, an algorithm was validated to detect motor fluctuations in patients with idiopathic PD, on the basis of inertial signals produced by a sensor attached to the patient’s waist. The high mean specificity and sensitivity values found for walking segments with 10 or more strides indicate good validity for this ON/OFF detection algorithm.

Results from patient 10 showed low sensitivity (0.56) when any walking segment was considered. However, sensitivity reached 0.82 for the longest segments. Similarly, the positive predictive value (PPV) for patient 15 was 0.48 when all the walking segments were considered, but reached 0.82 for the longest ones. Thus, the analysis of data from both patients required disregarding short walks since, in the first case, ON segments could be confused with OFF segments and, in the second case, false positives could arise. Regarding patient 12, PPV was 0.48 due to a low number of OFF segments (8), and a similar number of false OFF positives. However, specificity and sensitivity values—less dependent on the sample size—were much higher (>0.8).

Other researchers attempted to identify the patient’s motor status. However, up to our knowledge, all of them used algorithms validated in the laboratory setting [3-6], although in some studies patients performed natural or spontaneous activities [3,6]. Keijers et al [3] validated a neural network-based algorithm in a controlled environment with specificity and sensitivity values slightly higher than ours (~0.96). These higher values could be due to the fact that they used the same data set both in the training and the evaluation of the neural network. These authors, however, disregarded long gait episodes, which can actually be analyzed with our algorithm. It could thus be speculated that both algorithms could be complementary. Hoff et al [6] also reported validation results of motor fluctuations during natural activities. However, their specificity and sensitivity values were lower than ours, and those of Keijsers et al [3]. Patel et al [4] reported highly accurate results of monitoring motor fluctuations with accelerometers placed on 8 different parts of the body, in patients performing specific motor tasks. However, although such an approach is useful to estimate the severity of the symptoms, it is not suitable for monitoring the motor status during daily life activities. Additionally, unlike systems used by other researchers [3-6], our system consisted of only one device attached to the waist, which made it easily portable and comfortable.

The relevance of automatic detection of the motor status resides in providing accurate information for physicians to adjust medication schedules, and the possibility of real-time modifications of drug infusion rates, for example, in apomorphine or duodopa pumps. Thus, the infusion rate or bolus administration could be automatically increased upon detection of an OFF phase. The possibility of mapping a subject’s motor activity, and objectively determining the time spent in ON or OFF, may additionally promote the use of such detectors in clinical trials for a more reliable determination of subjects’ responses to experimental medication.

### Limitations

A limitation of our motor status (ON/OFF) detection algorithm derives from the fact that it is based on the analysis of patients’ movements while walking. Thus, the algorithm is unable to detect status changes when the patient is at rest. This may lead to the occurrence of long periods without motor information, which is especially critical since patients in OFF tend to stay still. In our opinion, further studies are required to detect OFF periods when patients are at rest (one of the objectives of the ongoing REMPARK project) [14]. However, since patients, even in moderate or advanced phases of the disease, walk more than 40 times per day [15,16], a system like ours, could still produce enough frequent detections. Although not continuous, detection of the motor status with our system could provide very useful information for a better clinical monitoring, since the ON/OFF periods usually last for 1 to 3 hours [17]. At present, no detection system at all is available to neurologists, and they have to rely on patients’ or caretakers’ reports to figure out the motor fluctuations. Certainly, a system that allows monitoring the motor status, even in an intermittent way, would be seen as a big step forward. Finally, it is worth mentioning that in our study the few OFF data recorded for some participants were more related to the short time they spent in OFF than to a lack of activity when they were in OFF.

The fact that the threshold has to be fixed for every individual patient requires an extra visit to calibrate the sensor. However, it is a simple procedure that in clinical practice would require the patient to take two short walks, one in OFF and one in ON. As the disease progresses, recalibration of the sensor may be necessary.

In technologically complex studies that require the use of technological research prototypes, the sample size is often small, a fact that undoubtedly poses limitations to generalization of results. We postulate that conducting further studies to evaluate the validity of the algorithm with larger samples and longer monitoring times is worth the effort.

### Conclusion

In conclusion, our results support the use of portable devices, easily accepted by patients with idiopathic PD for monitoring motor fluctuations in their habitual environment. The use of such devices would open the way to enhanced control of pharmacological therapy and to interactions with other electronic devices, such as drug infusion pumps.

## Abbreviations

CV: cross validation
IQR: interquartile range
PD: Parkinson’s disease
PPV: positive predictive value
SVM: support vector machine
